# Long-term self-reported health and disability after COVID-19 in public employees

**DOI:** 10.1186/s12889-022-14820-3

**Published:** 2022-12-21

**Authors:** Linda Ashman Kröönström, Johanna Krause, Simon B. Larsson, Robert Sigström, Katharina Stibrant Sunnerhagen

**Affiliations:** 1 Institute of Neuroscience and Physiology, Sahlgrenska Academy, University of Gothenburg, Gothenburg, Sweden; 2 Occupational and Physical Therapy Department, Sahlgrenska University Hospital, Gothenburg, Sweden; 3 Department of Infectious Diseases, Institute of Biomedicine, Sahlgrenska Academy, University of Gothenburg, Gothenburg, Sweden; 4 Department of Addiction and Dependency, Sahlgrenska University Hospital, Region Västra Götaland, Gothenburg, Sweden; 5 Department of Psychiatry and Neurochemistry, Institute of Neuroscience and Physiology, Sahlgrenska Academy, University of Gothenburg, Gothenburg, Sweden; 6 Department of Cognition and Old Age Psychiatry, Sahlgrenska University Hospital, Region Västra Götaland, Gothenburg, Sweden

**Keywords:** SARS-CoV-2, COVID-19, Post-acute COVID-19 syndrome, Rehabilitation, Disability, International Classification of Functioning, Disability and Health

## Abstract

**Background:**

Although the symptomatology has been assessed in multiple studies among persons recovering from coronavirus disease 2019 (COVID-19), less is known regarding long-term general health and disability. We aimed to assess long-term self-reported disability in public employees after predominantly mild COVID-19 in comparison with individuals who had negative COVID-19 polymerase chain reaction (PCR) test results.

**Methods:**

Public employees within Region Västra Götaland were offered tests to identify SARS-CoV-2 infection (*n* = 56,221) and were invited to complete an online survey that included the World Health Organization Disability Assessment Schedule. Questionnaires were sent out between January 26 and March 5, 2021. A total of 14,222 (25.3%) employees responded, of which 10,194 (18%) were included (women *n* = 8749, 85.8%). Of these, 7185 (70.5%) participants had a negative PCR test result (controls). A total of 1425 (14%) had a positive PCR result and were categorized in the sub-acute phase (4–12 weeks post COVID-19), and 1584 (15.5%) had a positive PCR test and were categorized in the post COVID-19 phase (> 12 weeks).

**Results:**

Fifty-two percent of controls rated disability of varying degrees, versus 73% and 64% of participants in the sub-acute and post COVID-19 phase, respectively. Being “emotionally affected” was the most frequently reported disability in the sub-acute phase, the post COVID-19 phase, as well as in controls. The proportion of participants reporting difficulties for 20–30 days was higher in the sub-acute phase than in the post COVID-19 phase (27.9% vs. 21.8%, *p* < 0.001) as well as in a comparison between participants in the post COVID-19 phase and controls (21.8% vs 14.2%, *p* < 0.001). Compared with controls, severe disability was more common in the post COVID-19 phase among both women (15.8% vs. 10.7%,), and men (9.8% vs. 6.8%).

**Conclusions:**

Disability was present in all groups; however, reported disability was greater in the sub-acute phase than in the post COVID-19 phase. The higher levels of disability reported in the COVID-19 patient population may indicate a persisting need for rehabilitation and recovery. In general, women reported a greater degree of disability than men in the sub-acute and post COVID-19 phases.

**Supplementary Information:**

The online version contains supplementary material available at 10.1186/s12889-022-14820-3.

## Background

Coronavirus disease 2019 (COVID-19) is caused by severe acute respiratory syndrome coronavirus 2 (SARS-CoV-2). By August 2022, nearly 600 million cases had been confirmed worldwide, with the equivalent number in Sweden approaching 2.6 million [1]. The severity (mild, moderate, severe, or critical) of COVID-19 [2] may influence the symptoms. Patients with mild COVID-19 are often able to recover at home whereas patients with critical COVID-19 require hospitalization [2]. A recent meta-analysis showed that 80% of individuals suffer at least one symptom 14–110 days following an acute infection [3]; however, methodological differences complicate the reporting of symptomatology [4]. Sub-acute COVID-19 has been defined as symptoms and abnormalities 4–12 weeks beyond acute COVID-19 [5]. The term post COVID-19 condition has been introduced by the World Health Organization (WHO) to define individuals with a history of probable or confirmed COVID-19 who have symptoms persisting for 3 months post-infection that cannot be explained by an alternative diagnosis [6].

The level of exposure to SARS-CoV-2 in society is variable. Some occupations require personnel to come in close contact with other individuals (such as patients) whereas in other occupations, personnel have the option to work from home. Health care workers, particularly those working with patients who have COVID-19, have been identified as a group at high risk of exposure [7]. Furthermore, occupations are sometimes dominated by a certain sex; therefore, women and men may have uneven levels of exposure to infection with SARS-CoV-2.

The International Classification of Functioning, Disability and Health (ICF) is a framework to describe health and health-related states [8]. Although the symptomatology has been extensively assessed in multiple studies among patients recovering from COVID-19 [3, 4], less is known regarding the aftermath of COVID-19 with respect to long-term health and disability. New disability has been found to be prevalent in over one-third of patients treated in an intensive care unit (ICU) owing to COVID-19 at 6 months after critical illness [9]. Health and disability are important to assess; correct knowledge is required to provide individualized rehabilitation, as well as to ensure that patients’ needs can be met in the health care system. Therefore, the purpose of the present study was to assess long-term health and disability among public employees after predominantly mild COVID-19 infection in comparison with individuals who had negative COVID-19 PCR test results (controls). Secondarily, we set out to assess the association of these factors in relation to sex and occupation.

## Participants and methods

### Study cohort

Beginning in March 2020, public employees working within in the Region Västra Götaland were offered a polymerase chain reaction (PCR) test to determine whether they had been infected with SARS-CoV-2. These tests were ordered by the employees nearest manager and the original rationale was to enable an immediate return to work among employees with a negative test result. Initially, testing was offered to employees in critical departments of hospitals. However, during 2020, the indication for testing was expanded and during the spring of 2020, all hospital employees as well as nursing home employees were included in testing. After the summer break of 2020, employees in additional services such as schools and social welfare, prison, and probation services were offered these tests. Participants received results of the PCR test via text to their mobile phone. We retrieved data regarding the PCR tests from a database. Between January 26 and March 5, 2021, we sent text messages in clusters of 5000–10,000 to all employees who had undergone testing before November 30, 2020. The text contained a link to the study website and contact information of the principal investigator. The study website included a full description of the study and a link to a questionnaire at https://www.1177.se, a Swedish online health care service driven by Inera AB on behalf of the regions of Sweden. It was not possible to submit the questionnaire without answering all questions. Two reminders were sent out, with the first sent 1 week after the first invitation. The study population has been described previously [10]. This study followed the Strengthening the Reporting of Observational Studies in Epidemiology (STROBE) reporting guidelines for observational studies [11].

**Fig. 1:**
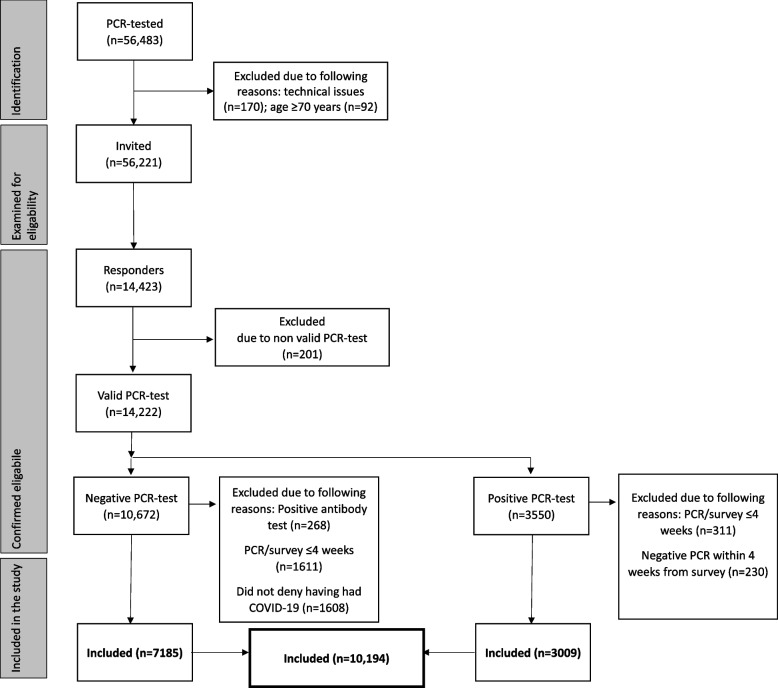
Flowchart of participant recruitment. PCR, polymerase chain reaction

In total, we identified 56,483 individuals in the database who had undergone at least one PCR test. An arbitrary exclusion criterion was applied because the study population included participants aged ≥ 70 years who were thought unlikely to be currently employed (*n* = 92). We also excluded 170 individuals for technical reasons. Of the 56,221 (99.5%) eligible participants who were sent the digital questionnaire, 14,423 (25.5%) replied. Of these, 14,222 participants (25.3% of those eligible) had a valid PCR test. However, of the 10,672 who had a negative PCR test, 2947 participants were excluded for the following reasons: positive antibody test result (*n* = 268, 2.5%); time from PCR to survey ≤ 4 weeks (*n* = 1611, 15.1%); and did not deny having had COVID-19 (*n* = 1608, 15.1%). Among the 3550 participants who had a positive PCR test, we excluded 311 (8.7%) with a time from PCR testing to the survey of ≤ 4 weeks and 230 (6.5%) with a negative PCR test within ≤ 4 weeks from the survey (Fig. 1).

### Measurements

We assessed the primary outcomes regarding long-term self-reported health and disability after predominantly mild COVID-19 using a questionnaire comprising questions such as how ill the participant was at the time of the PCR test and prior comorbidities at least during the past year. The questionnaire also included the World Health Organization Disability Assessment Schedule (WHODAS) questionnaire [12].

#### 12-item WHODAS 2.0

To assess health and disability, we used the Swedish version of the 12-item WHODAS 2.0 [12], which is a generic instrument developed based on and linked to the activity and participation component of the ICF [8]. WHODAS assesses an individual’s self-reported health and disability during the preceding 30 days. The respondent is instructed to assess the overall difficulty in performing a certain task. The short version consists of 12 items covering six domains (cognition, mobility, self-care, getting along, life activities, and participation), with responses ranging according to difficulty on a 5-point Likert scale, as follows: none, mild, moderate, severe, extreme/cannot do (0–4, respectively). The total score ranges from 0 (no disability) to 48 (complete disability). Items 13–15 assess the extent of difficulties during the preceding 30 days. The psychometric properties of the WHODAS have been evaluated in multiple countries and populations [13, 14], and general population norms are available [12].

### Definitions

The post-acute COVID-19 phase was divided into two groups as follows: the time from the PCR test was defined as the sub-acute phase (4–12 weeks beyond acute COVID-19), and the post COVID-19 phase (> 12 weeks beyond acute COVID-19) [6]. Occupations were classified into *major* groups according to the Swedish Standard Classification of Occupations 2012 [15], which in turn is based on the International Standard Classification of Occupations-08 [16]. Body mass index (BMI) was defined according to the WHO as follows: underweight < 18.5 kg/m^2^; normal weight 18.5–24.9 kg/m^2^; pre-obesity 25–29 kg/m^2^; obesity class I 30–34.9 kg/m^2^; obesity class II 35–39.9 kg/m^2^; and obesity class III ≥ 40 kg/m^2^ [17]. Participants reporting unlikely BMI values were not included in the analysis.

### Statistical methods

Data were analyzed using Statistical Package for Social Sciences (SPSS) 28.0 (IBM Corp., Armonk, NY, USA). Demographic data are presented as mean (standard deviation, SD). Regarding the WHODAS, because the data were not normally distributed, the data are presented as median (interquartile range [IQR]), and comparisons between groups were performed with the Mann–Whitney U test. A two-tailed *p*-value < 0.05 was considered to represent statistical significance. Data regarding the WHODAS were calculated according to the manual using simple scoring [12]. The total score of WHODAS items 1–12 was also classified into four groups according to degree of disability, as follows: none 0 points; mild 1–4; moderate 5–9; or severe 10–48 points [18]. In particular, we examined participants’ rating of various degrees of disability (1–48 points). Regarding WHODAS items 13–15, the number of days with difficulties during the preceding 30 days were divided into three groups, as follows: 0–9; 10–20; and 21–30 days.

## Results

Drop-out analysis of non-participants (*n* = 41,999) showed that included participants were older (45 ± 12 years vs. 42 ± 13 years, *p* < 0.001) and comprised a larger proportion of women (86.4% vs. 79.3%, *p* < 0.001). Based on aggregated data regarding PCR test positivity in the eligible population, we estimate that the response rate was approximately 50% among participants with a positive PCR test and 20% among those with a negative PCR test.

### Characteristics of the study population

The present study comprised 10,194 participants, of which most were women (*n* = 8749, 85.8%); and the mean age was 44.9 years (SD 12.1). Of these, 7185 (70.5%) participants had a negative PCR test, 1425 (14%) had a positive PCR test and were in the sub-acute phase, and 1584 (15.5%) had a positive PCR test and were in the post COVID-19 phase (Table 1).

**Table 1:** Characteristics of the study population according to sex, test results, and follow-up time (*n* = 10,194)

Participants	Negative PCR		Positive PCR 4–12 weeks		Positive PCR > 12 weeks	
	Women *n* = 6181	Men *n* = 1004	Women *n* = 1248	Men *n* = 177	Women *n* = 1320	Men *n* = 264
Time from PCR test to questionnaire, months	6.2 (2.5)	6.1 (2.5)	2.1 (0.6)	2.1 (0.6)	6.8 (2.9)	6.6 (2.8)
Age, years	45.2 (12.1)	43.7 (11.9)	44.7 (11.8)	42.9 (11.6)	44.7 (12.4)	43.8 (12.2)
BMI	26.2 (5.2) *	26.6 (4.4)	26.8 (5.3)	26.5 (4.0)	26.4 (5.3)	25.4 (4.5)
Nicotine use, yes, %						
Smoking	631 (10.2)	78 (7.8)	102 (8.2)	10 (5.6)	89 (6.7)	8 (3.0)
Snuff	584 (9.4)	241 (24.0)	132 (10.6)	46 (26.0)	132 (10.0)	74 (28.0)
Other tobacco free nicotine	192 (3.1)	17 (1.7)	43 (3.4)	1 (0.6)	33 (2.5)	3 (1.1)
Number of PCR tests	2.3 (1.11)	2.1 (1.07)	2.7 (1.02)	2.5 (1.02)	2.1 (1.07)	1.9 (1.04)
Ill at the time of PCR test, %						
No symptoms	810 (13.1)	167 (16.6)	103 (8.3)	13 (7.3)	57 (4.3)	10 (3.8)
Mild symptoms (not bedridden)	4172 (67.5)	691 (68.8)	431 (34.5)	76 (42.9)	441 (33.4)	103 (39)
Bedridden (at least 4 h/day)	1147 (18.6)	141 (14)	668 (53.5)	85 (48)	765 (58)	139 (52.7)
Needed care	52 (0.8)	5 (0.5)	38 (3.0)	2 (1.1)	49 (3.7)	7 (2.7)
Hospitalized	0 (0)	0 (0)	8 (0.6)	1 (0.6)	8 (0.6)	5 (1.9)
Self-reported illness prior to COVID-19 test, yes, %	3102 (50.2)	451 (44.9)	637 (51.0)	76 (42.9)	612 (46.4)	111 (42.0)
Asthma	646 (10.5)	82 (8.2)	141 (11.3)	16 (9.0)	133 (10.1)	19 (7.2)
Allergy	1350 (21.8)	201 (20.0)	288 (23.1)	41 (23.2)	274 (20.8)	52 (19.7)
Diabetes type I	60 (1.0)	19 (1.9)	7 (0.6)	0 (0)	14 (1.1)	5 (1.9)
Diabetes type II	109 (1.8)	21 (2.1)	26 (2.1)	3 (1.7)	26 (2.0)	6 (2.3)
Hypertension	678 (11.0)	132 (13.1)	127 (10.20)	24 (13.6)	131 (9.9)	36 (13.6)
Other cardiovascular disease	138 (2.2)	38 (3.8)	26 (2.1)	4 (2.3)	26 (2.0)	9 (3.4)
Mental disorder	974 (15.8)	130 (12.9)	213 (17.1)	12 (6.8)	160 (12.1)	21 (8.0)
Thyroid illness	473 (7.7)	19 (1.9)	107 (8.6)	2 (1.1)	97 (7.3)	6 (2.3)
Other autoimmune disease	453 (7.3)	49 (4.9)	76 (6.1)	12 (6.8)	85 (6.4)	11 (4.2)
Occupation, % **						
Manager	375 (6.1)	86 (8.6)	47 (3.8)	15 (8.5)	70 (5.3)	16 (6.1)
Health care professional requiring college education	1602 (25.9)	304 (30.3)	320 (25.6)	55 (31.1)	434 (32.9)	101 (38.3)
Care worker	2243 (36.3)	259 (25.8)	620 (49.7)	58 (32.8)	580 (43.9)	70 (26.5)
Other profession requiring college education	1559 (25.2)	215 (21.4)	216 (17.3)	26 (14.7)	176 (13.3)	46 (17.4)
Other	390 (6.3)	132 (13.1)	44 (3.5)	23 (13.0)	57 (4.3)	28 (10.6)
Unknown	12 (0.2)	8 (0.8)	1 (0.1)	0 (0)	3 (0.2)	3 (1.1)
Place of residence, %						
Urban areas of Gothenburg	5095 (82.4)	877 (87.4)	1037 (83.0)	157 (88.7)	1121 (84.9)	232 (87.9)
Rural	1031 (16.7)	108 (10.8)	208 (16.7)	20 (11.3)	186 (14.1)	27 (10.2)
Other parts of Sweden or unknown	55 (0.9)	19 (1.9)	3 (0.2)	0 (0)	13 (1.0)	5 (1.9)
Total score of WHODAS, points	1 [0, 4]	0 [0, 4]	4 [1, 10]	1 [0, 5]	2 [0, 6]	1 [0, 5]

Data are n (%), mean (standard deviation) or median [interquartile range]

*PCR* Polymerase chain reaction, *COVID-19* Coronavirus disease 2019, *BMI* Body mass index

^*^n = 6177 owing to four unlikely/extreme values

^**^ Examples of included occupations: Manager; Health care occupations requiring college education (medical doctor, nurse, dentist, physiotherapist, veterinarian); Care workers (assistant nurse, support assistant, habilitation staff, personal assistant); Other professions requiring college education (teacher, social secretary, lawyer, and engineer); Other (firefighter, chef)

### WHODAS

**Fig. 2:**
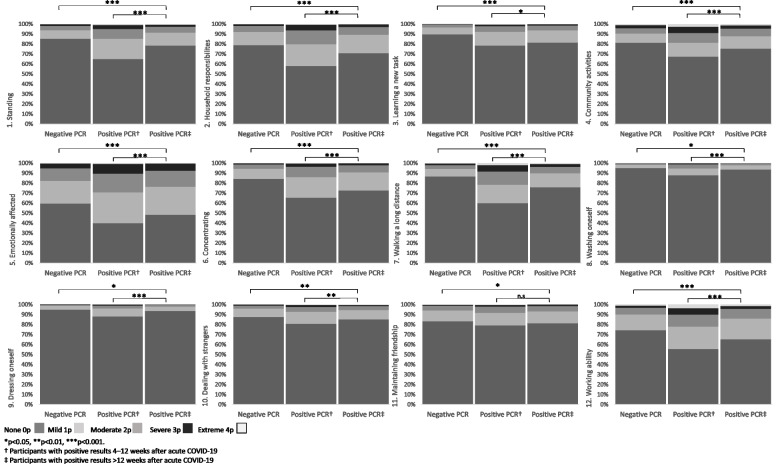
Disability according to WHODAS (items 1–12), test results and follow-up time after PCR testing. WHODAS, World Health Organization Disability Assessment Schedule; PCR, polymerase chain reaction; n.s., non-significant. *n* = 10,194 (negative PCR test *n* = 7185; positive PCR test 4–12 weeks *n* = 1425; positive PCR > 12 weeks *n* = 1584)

**Fig. 3:**
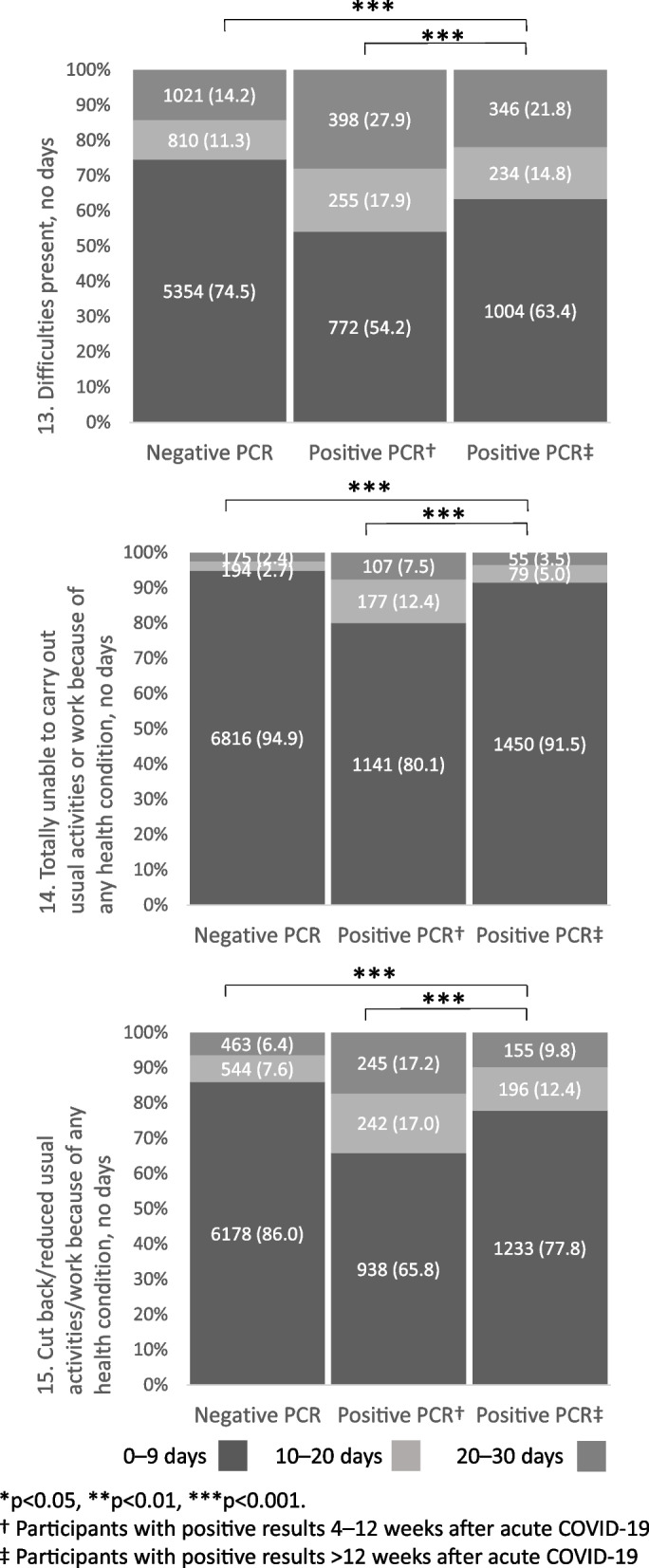
Number of days with difficulties according to test results and follow-up time after PCR testing. WHODAS, World Health Organization Disability Assessment Schedule; PCR, polymerase chain reaction; n.s., non-significant. *n* = 10,194 (negative PCR test *n* = 7185; positive PCR test 4–12 weeks *n* = 1425; positive PCR > 12 weeks *n* = 1584). Numbers in parentheses are %

**Fig. 4:**
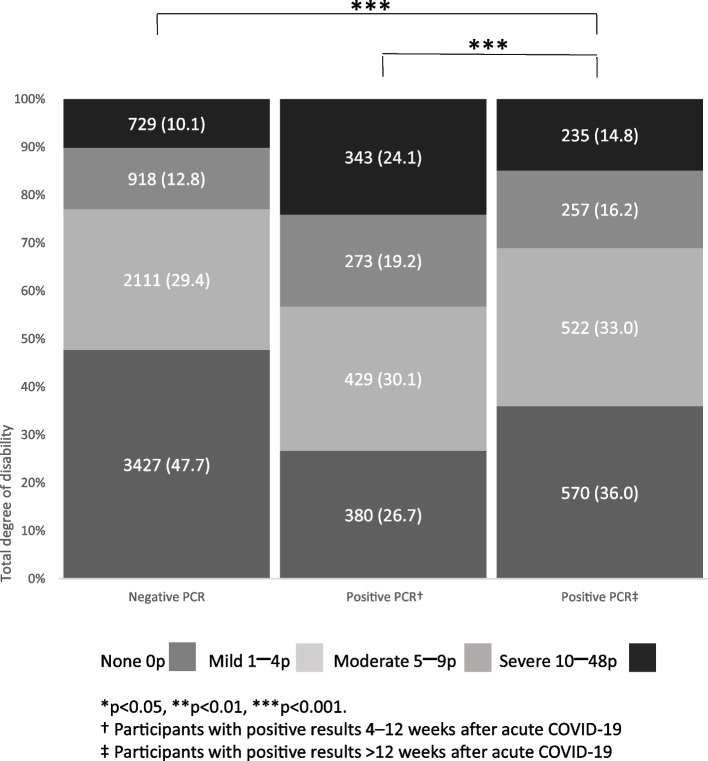
Total degree of disability according to WHODAS, test results and follow-up time after PCR testing. WHODAS, World Health Organization Disability Assessment Schedule; PCR, polymerase chain reaction; n.s., non-significant. *n* = 10,194 (negative PCR test *n* = 7185; positive PCR test 4–12 weeks *n* = 1425; positive PCR > 12 weeks *n* = 1584). Numbers in parentheses are %

Figures 2 and 3 present disability according to individual WHODAS items. Participants in the post COVID-19 group reported greater disability than controls regarding all 15 items, and those in the sub-acute phase reported greater disability than participants in the post COVID-19 group for 14 WHODAS items. The pattern was the same regarding total WHODAS score (Fig. 4). “Emotionally affected” was the most frequently reported disability item (Fig. 2). In participants reporting disability, the proportions of participants being “emotionally affected” were 59.9% in the sub-acute phase, 51.6% in the post-acute phase, and 40.3% in controls (Supplementary Table 1, Additional File 1). The proportion of participants reporting difficulties for 20–30 days was higher in the sub-acute phase than in the post COVID-19 phase (27.9% vs. 21.8%, *p* < 0.001) as well as in comparison between participants in the post COVID-19 phase and controls (21.8% vs. 14.2%, *p* < 0.001). Furthermore, participants in the post COVID-19 phase reported having a reduced number of days in which they could participate in normal activities or work to a greater extent than controls (9.8% vs. 6.4%, *p* < 0.001); however, this was reported to a lesser extent by the former group than by participants in the sub-acute phase (9.8% vs. 17.2%, *p* < 0.001) (Fig. 3).

Regarding the analysis according to sex, women reported a higher degree of disability than men in 5 items among controls, in 10 items among participants in the sub-acute phase, and in 7 items among participants in the post COVID-19 phase for WHODAS items 1–12 (Supplementary Fig. 1, Additional File 1). Regarding WHODAS items 13–15, women in all three groups reported more days of disability than men (Supplementary Fig. 2, Additional File 1) as well as a greater total degree of disability (Supplementary Fig. 3, Additional File 1). Furthermore, 10.7% of women and 6.8% of men with negative PCR tests reported the total degree of disability as severe as opposed to 25.5% of women and 14.1% of men in the sub-acute phase and 15.8% of women and 9.8% of men in the post COVID-19 phase (Supplementary Fig. 3, Additional File 1).

Regarding occupations, care workers constituted the largest proportion of participants in this study. In all five occupation groups, participants in the post COVID-19 phase reported greater disability than controls. Participants in the sub-acute phase within the occupations of manager, health care professional requiring a college education, and care workers reported greater disability than participants in the post COVID-19 phase (*p* < 0.001) (Supplementary Fig. 4, Additional File 1).

## Discussion

We report our study findings regarding the long-term effects on health and disability among public employees after predominantly mild COVID-19. We found that the majority of participants reported disability, but the prevalence was higher among participants in the sub-acute phase (73%) and post COVID-19 phase (64%) than among negative controls (52%). There was a lower prevalence of disability among participants in the post COVID-19 phase than in the sub-acute phase. This could be interpreted as an indication of a natural course of recovery. Conversely, in a sample of symptomatic patients from the general population (*n* = 197) the number of patients reporting full recovery at 12 vs. 18 months was almost equivalent (39% and 38% respectively) [19]. Furthermore, in hospitalized patients due to COVID-19 the proportion of patients who reported full recovery at 5 months vs. 1 year was unchanged [20].

The clinical case definition published by the WHO of post COVID-19 condition includes the phrase “a history of probable or confirmed COVID-19” [21]. As the present study did not include a clinical examination of participants; disability reported may result from causes other than COVID-19. In the present study, 15.8% of women and 9.8% of men reported severe disability in the post COVID-19 phase as opposed to participants with negative PCR tests reporting severe disability (10.7% of women and 6.8% of men). Disability has been assessed in critically ill patients with COVID-19 [9, 22]. Hodgson et al. [9] found new disabilities at 6 months after critical illness in over one-third of patients treated in the ICU. Disability has not been previously evaluated with WHODAS in patients with predominantly mild COVID-19; further, comparing critically ill patients with patients who have predominantly mild COVID-19 may be problematic.

We found a greater level of disability in women than men. These results are in line with a previous report in a normative population showing that women report slightly greater disability than men, according to the WHODAS [18]. Analysis according to sex regarding WHODAS items 1–12 showed that women more often than men reported disability in the sub-acute phase as compared with their counterparts in the post COVID-19 phase or controls. In the present study, 46.3% of women and 31.1% of men in the sub-acute phase, and 35.6% of women and 29.9% of men in the post COVID-19 phase reported disability of varying degrees for the WHODAS item “Working ability.” These proportions are in comparison with the 25.7% of women and 25.8% of men among controls who reported disability, and a possible need for rehabilitation and recovery to regain their ability to work. In the Swedish population, risk factors for recurrent sick leave owing to COVID-19 have been found to be female sex, prior sick leave, older age [23], and the severity of COVID-19 [24]. The large predominance of women (85.8%) in the present study could be explained by the sex difference in Swedish public employees (71.3% women) [25].

The WHODAS covers all areas of the ICF [8]. There is no cut off value for significant disability with respect to the WHODAS. However, the grading of WHODAS into mild, moderate, or severe disability has previously been used in normative data [18] and in other patient groups, such as those with stroke [26]. Participants having persisting symptoms not explained by objective findings such as a verified positive PCR test may be explained by the fact that other factors such as prior illness, comorbidities, or level of physical function influence health and disability. In the present study, the proportion of respondents with self-reported illness prior to COVID-19 ranged from 42%–51%. In data from an Australian normative population, people with mental health disorders reported greater degrees of disabilities (mean 6.3 ± 7.1) than people with physical disorders (mean 4.3 ± 6.1) [18]. The WHODAS item”How much have you been emotionally affected by your health problems” was the most frequently reported disability in participants with positive PCR results as well as controls. The WHODAS item “How much of a problem do you have joining in community activities (festivities, religious or other activities) in the same way as anyone else can?” may have been difficult to respond to as questionnaires were administered during a period when traveling, socializing, and workplace restrictions were in place in Sweden.

Disability is an umbrella term for impairment, activity limitation, and restricted participation [12]; therefore, it is important to detect disability to be able to provide individualized multi-disciplinary rehabilitation to those in need [27]. The results of the present study showed that being emotionally affected by COVID-19 was the most commonly reported disability. The complexity, heterogenous symptomatology, and disability vary. Each individuals’ needs are determinants of how limited a person may be owing to new disability and which requirements for rehabilitation each individual may have. Future studies with an even longer perspective are needed to assess disability, the natural course of recovery, and the potential need for individualized rehabilitation after COVID-19.

### Strengths and limitations

The strengths of the present study include the relatively large sample size and the inclusion of a control group for comparison. Answers to questionnaire items were mandatory so there were no missing variables. The questionnaire contained one question regarding if the participants themselves thought that they had had COVID-19. This question helped us exclude participants from the control group who thought that they had had COVID-19, and therefore improving the selection of participants to the control group. We acknowledge the following limitations in the present study. First, the response rate was 25%, which is a limitation that entails a loss of power; however, approximately 50% of participants with a positive PCR test result were included in the study. Two reminders about the questionnaire were sent to increase participation. Antibody tests were offered to employees on a voluntary basis outside of the study. Therefore, we did not have data regarding this in all participants. Public employees within Region Västra Götaland were urged to get tested whenever they experienced symptoms (even mild ones); however, if the person nonetheless chose not to get tested and then reported “not having had COVID-19” they could have been misclassified into the group of persons with negative PCR results (controls). Asymptomatic persons could have been misclassified as controls and symptoms and disability in this group may therefore have been underestimated. Testing was not established in all work places until the fall of 2020 and access in the community was limited until summer 2020. Thus, some participants may have had unconfirmed COVID-19. Also, although we had access to all tests ordered by participants’ employers, access to tests ordered in routine care was limited primarily in one part of Region Västra Götaland (Skaraborg). Thus, some participants may have had confirmed COVID-19 without our knowledge. However, by also asking participants if they thought they had had COVID-19, we believe that we minimized the number of negative controls who had in fact had COVID-19. Presumably, the population with symptoms were more likely to complete the questionnaire. Furthermore, the study participants mainly comprised women of working age; therefore, our findings may not be generalizable to other populations. The time from PCR testing to completing the questionnaire varied; a longer follow-up increases the risk of recall bias regarding the degree of acute disease. Finally, the WHODAS is a generic instrument that has not been validated for use in patients with COVID-19; however, the WHODAS has been used previously to describe disability in patients with COVID-19 treated in the ICU [9, 22].

## Conclusion

In the present study, disability was present in all groups. However, disability was greater in the sub-acute phase than in the post COVID-19 phase. The higher levels of disability reported in the COVID-19 patient population may indicate a persisting need for rehabilitation and recovery. In general, women reported a greater degree of disability than men in the sub-acute and post COVID-19 phase.

## Supplementary Information

**Additional file 1: Supplementary Table 1.** The number of participants reporting disability (mild, moderate, severe, extreme) on each WHODAS item (1–12). **Supplementary Figure 1.** Disability according to WHODAS, sex, test results and follow-up time after PCR testing. **Supplementary Figure 2.** Difference in number of days with difficulties according to sex, test results and follow-up time. **Supplementary Figure 3.** Total degree of disability according to WHODAS, sex, test results and follow-up time. **Supplementary Figure 4**. Total degree of disability with respect to occupation, test results, and follow-up time.

## Data Availability

The datasets generated and/or analyzed during the current study are not publicly available for ethical reasons but are available from the corresponding author on reasonable request as well as with valid ethical approval.

## Abbreviations

COVID-19: Coronavirus disease 2019
SARS-CoV-2: Severe acute respiratory syndrome coronavirus 2
ICF: International Classification of Functioning, Disability and Health
ICU: Intensive care unit
PCR: Polymerase chain reaction
STROBE: Strengthening the Reporting of Observational Studies in Epidemiology
WHODAS: World Health Organization Disability Assessment Schedule
BMI: Body mass index
WHO: World Health Organization
SD: Standard deviation
IQR: Interquartile range
