# Supplementary material for: Long-term self-reported health and disability after COVID-19 in public employees
Source: BMC Public Health. 2022 Dec 21;22:2400. doi: 10.1186/s12889-022-14820-3 (PMC9768407; doi:10.1186/s12889-022-14820-3)
Supplement: Supplementary file 1 — Additional file 1: Supplementary Table 1. The number of participants reporting disability (mild, moderate, severe, extreme) on each WHODAS item (1–12). Supplementary Figure 1. Disability according to WHODAS, sex, test results and follow-up time after PCR testing. Supplementary Figure 2. Difference in number of days with difficulties according to sex, test results and follow-up time. Supplementary Figure 3. Total degree of disability according to WHODAS, sex, test results and follow-up time. Supplementary Figure 4. Total degree of disability with respect to occupation, test results, and follow-up time. [file 12889_2022_14820_MOESM1_ESM.docx]

**Additional file 1.**

1. **Results**

The number of participants reporting disability on each of the 12 WHODAS items are shown in Supplementary Table 1. Results of disability in women and men according to WHODAS (items 1–12, items 13–15), and the total degree of disability are shown in Supplementary Figures 1–3. The total degree of disability according to WHODAS with respect to occupation is presented in Supplementary Figure 4.

**Supplementary Table 1. The number of participants reporting disability (mild, moderate, severe, extreme) on each WHODAS item (1–12)**

| WHODAS item | Negative PCR  n=7185 | Positive PCR  4–12 weeks  n=1425 | Positive PCR  >12 weeks  n=1584 |
| --- | --- | --- | --- |
| 1. Standing | 1051 (14.6) | 497 (34.9) | 342 (21.6) |
| 1. Household responsibilites | 1521 (21.2) | 597 (41.9) | 461 (29.1) |
| 1. Learning a new task | 744 (10.4) | 308 (21.6) | 292 (18.4) |
| 1. Community activities | 1337 (18.6) | 464 (32.6) | 387 (24.4) |
| 1. Emotionally affected | 2897 (40.3) | 854 (59.9) | 818 (51.6) |
| 1. Concentrating | 1126 (15.7) | 493 (34.6) | 432 (27.3) |
| 1. Walking a long distance | 964 (13.4) | 571 (40.1) | 383 (24.2) |
| 1. Washing oneself | 358 (5.0) | 174 (12.2) | 100 (6.3) |
| 1. Dressing oneself | 367 (5.1) | 169 (11.9) | 102 (6.4) |
| 1. Dealing with strangers | 900 (12.5) | 276 (19.4) | 237 (15.0) |
| 1. Maintaining friendship | 1201 (16.7) | 297 (20.8) | 297 (18.8) |
| 1. Working ability | 1843 (25.7) | 633 (44.4) | 549 (34.7) |

Data are number of (%).

WHODAS, World Health Organization Disability Assessment Schedule; PCR, polymerase chain reaction.

**Supplementary Figure 1. Disability according to WHODAS, sex, test results and follow-up time after PCR testing.**

WHODAS, World Health Organization Disability Assessment Schedule; PCR, polymerase chain reaction; n.s., non-significant.

n=10,194 (negative PCR test women n=6181, men n=1004; positive PCR test 4–12 weeks women n=1248, men n=177; positive PCR >12 weeks women n=1320, men n=264).

**Supplementary** **Figure 2. Difference in number of days with difficulties according to sex, test results and follow-up time.**

WHODAS, World Health Organization Disability Assessment Schedule; PCR, polymerase chain reaction; n.s., non-significant.

n=10,194 (negative PCR test women n=6181, men n=1004; positive PCR test 4–12 weeks women n=1248, men n=177; positive PCR >12 weeks women n=1320, men n=264).

**Supplementary Figure 3. Total degree of disability according to WHODAS, sex, test results and follow-up time.**

WHODAS, World Health Organization Disability Assessment Schedule; PCR, polymerase chain reaction; n.s., non-significant.

n=10,194 (negative PCR test women n=6181, men n=1004; positive PCR test 4–12 weeks women n=1248, men n=177; positive PCR >12 weeks women n=1320, men n=264).

Numbers in parentheses are %.

**Supplementary Figure 4**. **Total degree of disability with respect to occupation, test results, and follow-up time.**

PCR, polymerase chain reaction; n.s., non-significant.

n=10,194 (negative PCR test women n=6181, men n=1004; positive PCR test 4–12 weeks women n=1248, men n=177; positive PCR >12 weeks women n=1320, men n=264)
